# Fibula Graft Cutting Devices: Are 3D-Printed Cutting Guides More Precise than a Universal, Reusable Osteotomy Jig?

**DOI:** 10.3390/jcm9124119

**Published:** 2020-12-20

**Authors:** Simon Meyer, Jan-Michaél Hirsch, Christoph S. Leiggener, Bilal Msallem, Guido R. Sigron, Christoph Kunz, Florian M. Thieringer

**Affiliations:** 1 Clinic of Oral and Cranio-Maxillofacial Surgery, University Hospital Basel, CH-4031 Basel, Switzerland; simon.meyer@usb.ch (S.M.); christoph.kunz@usb.ch (C.K.); ft@swiss-mam.ch (F.M.T.); 2 Medical Additive Manufacturing Research Group (Swiss MAM), Department of Biomedical Engineering, University of Basel, CH-4123 Allschwil, Switzerland; guido.sigron@usb.ch; 3 Department of Surgical Sciences, Oral and Maxillofacial Surgery, Uppsala University, SE-751 85 Uppsala, Sweden; Jan.Hirsch@surgsci.uu.se; 4 Department of Research & Development Public Dental Services Folktandvården AB, SE-118 27 Stockholm, Sweden; 5 Clinic of Oral and Cranio-Maxillofacial Surgery, Kantonsspital Aarau, CH-5001 Aarau, Switzerland; christoph.leiggener@ksa.ch

**Keywords:** cranio-maxillofacial surgery, mandibular reconstruction, fibula, osteotomy, tissue transplantation, surgical guides

## Abstract

Individual cutting guides for the reconstruction of lower jaw defects with fibular grafts are often used. However, the application of these osteotomy tools is costly and time intensive. The aim of this study was to compare the precision of osteotomies using a 3D-printed guide with those using a universal, reusable, and more cost-efficient Multi-Use Cutting Jig (MUC-Jig). In this non-blinded experimental study, 10 cranio-maxillofacial surgeons performed four graft removals each in a randomized order using the same osteotomy angle, both proximally (sagittal cut) and distally (coronal cut), of a graft (45°, 30°, 15°, or 0°), first with the MUC-Jig then with the 3D-printed cutting guide. The 40 fibula transplants (Tx) of each method (n = 80) were then analyzed concerning their Tx length and osteotomy angles and compared to the original planning data. Furthermore, the surgeons’ subjective perception and the duration of the two procedures were analyzed. The mean relative length and mean relative angle deviation between the MUC-Jig (−0.08 ± 1.12 mm; −0.69° ± 3.15°) and the template (0.22 ± 0.90 mm; 0.36° ± 2.56°) group differed significantly (*p* = 0.002; *p* = < 0.001), but the absolute deviations did not (*p* = 0.206; *p* = 0.980). Consequently, clinically comparable osteotomy results can be achieved with both methods, but from an economic point of view the MUC-Jig is a more cost-efficient solution.

## 1. Introduction

The reconstruction of mandible defects using a free fibula graft was first described by Hidalgo in 1989 and is nowadays an established method in cranio-maxillofacial (CMF) surgery [1]. Initially, the procedure was performed freehand, and the surgeons’ experience, routine, and skills were essential factors that potentially influenced the outcome, since the shaping of the fibula, as well as the bending of the plates, had to be performed intraoperatively [2].

In the years that followed, pre-bent reconstruction plates [3,4], electron beam melting manufactured reconstruction plates [5], prefabricated templates [6], and osteotomy guides [7,8,9] were also described and applied.

Once computers became a part of everyday surgical practice, jaw reconstructions started to be performed using virtual surgical planning (VSP) [10,11,12], which is now state of the art in may centers. Individual cutting guides and preformed reconstruction plates have been produced on the basis of digital 3D data, since then to assist the surgeon during the operation to perform osteotomies at the correct angle and in the right place, as well as to reconstruct the shape and position of the mandible according to the planned parameters. On the one hand, this improves surgical workflow in the operating theater, and on the other hand leads to better results compared to the freehanded method, especially in complex cases [13,14,15]. In addition to the advantages of better surgical preparation [16,17,18] and a reduction in the operation time [15,19,20], this method has disadvantages, such as dependence on industry and loss of time due to outsourced planning, production, and shipping of the template and the resulting high costs. Subsequently, template-guided surgery remains more expensive than freehand surgery [20].

Reports of reusable, more economical, and universal saw gauges have been published in recent years [7,8,9], one of which is the Multi-Use Cutting Jig (MUC-Jig); its use was described in an earlier publication of our research group [21]. Reusable osteotomy tools are more cost-efficient alternatives to a 3D-printed cutting guide. Due to their reusability and their universal applicability for all patients, they have only one-time upfront costs. They can be used with or without pre-bent plates.

The aim of this non-blinded experimental in vitro study was to test the hypothesis that the osteotomy results reached with a MUC-Jig do not significantly differ from the results reached with a 3D-printed cutting guide, neither in the length nor in the angle conversion of the segments.

## 2. Material and Methods

### 2.1. The Multi-Use Cutting Jig (MUC-Jig)

**Figure 1:**
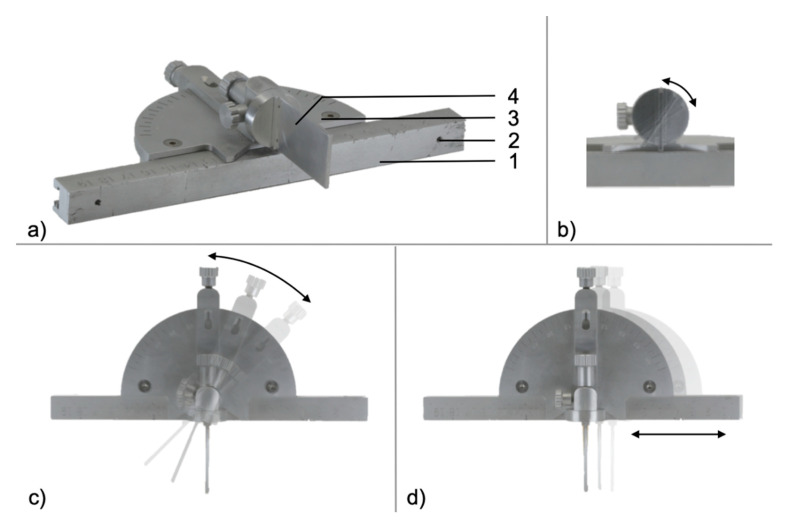
Diagonal view of Multi-Use Cutting Jig (MUC-Jig) (**a**). Ruler-rail (1), which can be attached to the fibula with two standard screws (2). The angle gauge (3) can be adjusted vertically (**b**) and horizontally (**c**) and can be moved along the ruler-rail (**d**). The stop (4) guides the saw blade intraoperatively over a distance of 25 mm, which provides a high degree of stability.

The self-developed MUC-Jig is a power miter saw manufactured in aluminum fabricated by CNC (computer numerical control) manufacturing. It is a sophisticated tool that allows a fast and accurate bone cut in a fibula osteomyocutaneous graft at selected angles and defined points. The power miter saw was initially invented by Ed Niehaus (1964) but was never patented. The original power miter saw makes cuts by pulling a rotating circular blade down onto a workpiece, while the MUC-Jig saw uses an oscillating saw blade (Colibri II, DePuy Synthes, Oberdorf, Switzerland), directed and supported by a stop. It is attached intraoperatively to the lateral side of the fibula with screws inserted through two drill holes in the ruler rail (Figure 1a). The angle stop is attached to the ruler-rail. It is used to define the cutting direction in the horizontal (coronal plane) and vertical (sagittal plane) orientation (Figure 1b,c). Finally, it is adjusted along the ruler-rail to the desired location on the fibula bone (Figure 1d).

### 2.2. Three-Dimensional-Printed Cutting Guide

**Figure 2:**
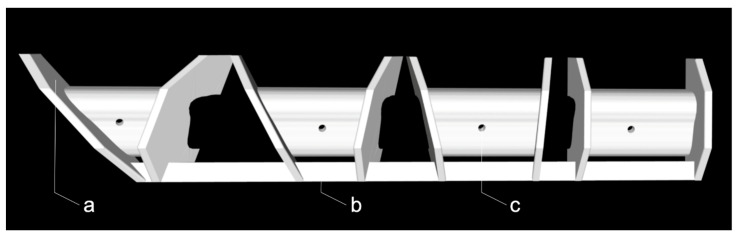
The 3D-printed cutting guide used in this study with simple stops (**a**), a connector to define the distance between the graft sites (**b**), and holes for screw fixation (**c**) per segment.

The cutting guide used in this experimental study was digitally planned (Mimics Innovation Suite, v. 20, Materialise, Leuven, Belgium) and made from a 3D-printer (Objet30 Prime v. 3, Stratasys, Ltd., Eden Prairie, MN, USA) using transparent biocompatible material with dissolvable support structures (MED610 with SUP706, Stratasys, Ltd., Eden Prairie, MN, USA) (Figure 2).

### 2.3. Study Model

**Figure 3:**
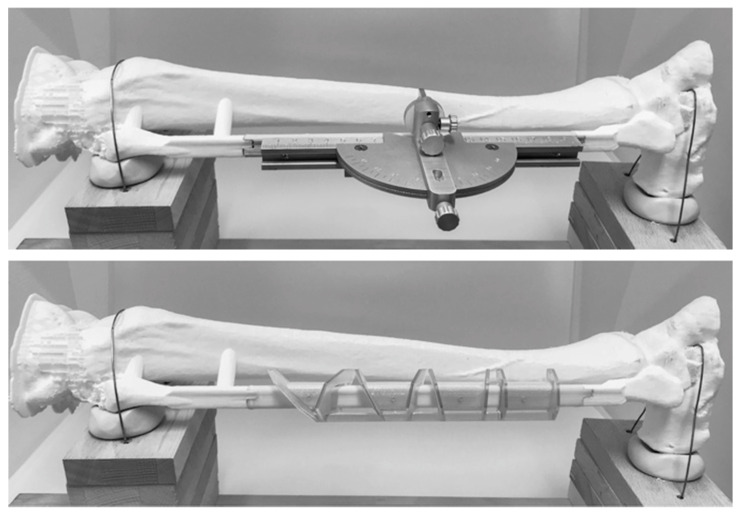
Study setup with the MUC-JIG (**top**) and the 3D-printed cutting guide (**bottom**).

A central axial cross-section of a fibula was digitally extruded using the medical software Mimics Innovation Suite v. 20 (Materialise, Leuven, Belgium) to create a standardized fibula model so that morphologically comparable results could be achieved regardless of the exact extraction location. The fibula was exchangeable and attached to a tibia model at both ends, which itself was fixed to a plate. The aim was to simulate the spatially limited intraoperative conditions (Figure 3). Both models were printed with a MakerBot Replicator+ 3D-printer from true white polylactic acid (PLA) filament with a diameter of 1.75 mm (MakerBot Industries, NY, USA).

### 2.4. In Vitro Surgery

**Figure 4:**
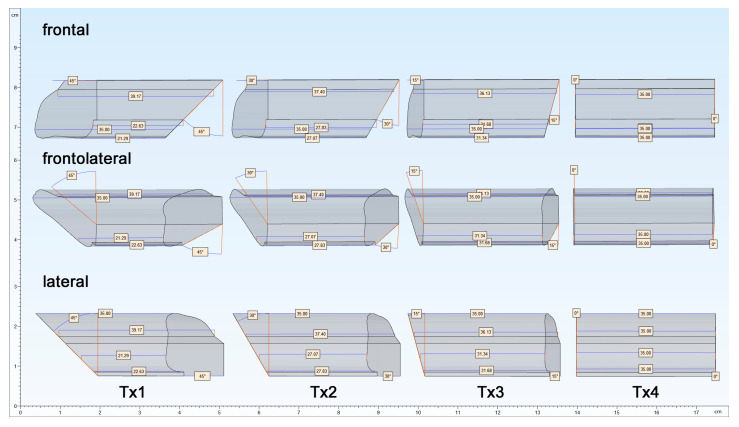
Original planning parameters, to be realized with both methods, with Tx1 (proximal) to Tx4 (distal) in frontal, frontolateral, and lateral views.

Ten CMF surgeons (five consultants and five residents) performed eight different osteotomies, first using the MUC-Jig and then using the 3D-printed guide**.** Thus, four different test sequences were performed with each of the two methods in a randomized order by each surgeon; the osteotomies were carried out on one segment, yet both proximally (sagittal plane) and distally (coronal plane) at the same angle (TX: 45°, TX2: 30°, TX3: 15° or TX4: 0°) but in different planes (Figure 4). Thereby, the 10 surgeons performed 160 osteotomies, leading to 80 fibula segments, equivalent to 40 with each method.

Before the operation, the surgeons were instructed to use the following tools: pencil (Write-4-All Permanent Marker, size F, black, STABILO International GmbH, Heroldsberg, Germany), stainless steel ruler (Lineal 50 cm Stahl, Veto, Alphen aan den Rijn, The Netherlands), digital planning with angle and length data of the four transplants (Mimics Innovation Suite, v. 20, Materialise, Leuven, Belgium) and the saw system (Colibri II, Synthes, DePuy Synthes, Oberdorf, Switzerland) with the AO/ASIF Quick Coupling (05.001.250, DePuy Synthes, Oberdorf, Switzerland), a 1.1 mm drill (310.110, DePuy Synthes, Oberdorf, Switzerland), as well as an Oscillating Saw Attachment (532.021, DePuy Synthes, Oberdorf, Switzerland) with a saw blade (532.064, DePuy Synthes, Oberdorf, Switzerland). The surgeons were also given instructions on the handling of the MUC-Jig and the 3D-printed cutting guide**.** Both osteotomy tools were placed on the lateral side of the fibula using screws (Spax 2 × 12 mm Senkkopf, SPAX International GmbH & Co. KG, Ennepetal, Germany). The osteotomies were then performed according to an intervention protocol, using the MUC-Jig first, followed by the 3D-printed cutting guide.

### 2.5. Evaluation

#### 2.5.1. Participant Information

The following variables were recorded using a digital questionnaire (Google Forms, Google Inc., Mountain View, CA, USA): sex, age, experience in CMF surgery since double licensure, and the number of performed and assisted fibula transplantations.

#### 2.5.2. Segment Analysis

**Figure 5:**
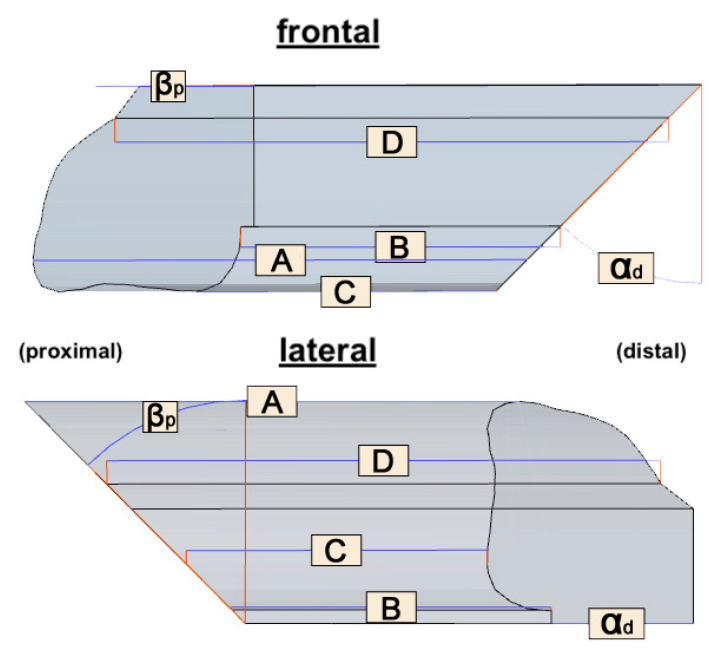
Assessed lengths (A: ventral, B: dorsal, C: lateral, D: medial) and varying angles from 45° to 0° (β_p_: sagittal plane, α_d_: coronary plane α_d_) of each transplant from frontal (**top**) and lateral (**bottom**). The fixed angles (α_p_: coronary plane, β_pd_: sagittal plane) with 0° are not shown in this figure.

The segments were analyzed analogously using a caliper gauge (Elektronischer Digital-Messschieber Din 862, Vogel, Kevelaer, Germany) and a protractor (LUX Winkelmesser, Lux GmbH & Co. KG, Wermelskirchen, Germany). Each segment was evaluated according to the length (A, B, C, D) at defined locations. The osteotomy directions were measured in two planes (α and β) proximally (p) and distally (d) (Figure 5). Each measurement was carried out in triplicate.
Segment length locations Osteotomy anglesA: ventral most C: lateral most α_p_ and α_d_: angle in the coronal planeB: dorsal most D: medial most β_p_ and β_d_: angle in the sagittal plane

#### 2.5.3. Intervention Time

During the graft removal, the following variables were gathered using video tapes of the operation, filmed with an overhead camera (iPhone 7, Apple, Cupertino, USA): the time used for preparation and the duration of the osteotomies.

The following steps were counted as preparation time (t_prep_):

- MUC-Jig: The time required for the attachment to the fibula, setting the fence before each cut, and for the removal of the cutting jig.
- Cutting guide: The time required to fix the guide to the fibula with screws and for the removal of the cutting guide.

The time for the osteotomy (t_ost_) resulted from the difference between the total duration (t_total_) and the preparation time (t_prep_).

#### 2.5.4. Surgeons’ Subjective Perception of the Osteotomies

The surgeons’ experiences were recorded by means of a digital questionnaire (Google Forms, Google Inc., Mountain View, CA, USA) after the osteotomies had been performed. The surgeons were asked to rate, on a visual analogue scale (VAS), the degree of subjective perceived assistance provided by the tool during the procedure, the difficulty of performing the osteotomies, the difficulty of transferring the cutting angle and the transplant length, their satisfaction with the result, and their satisfaction with their performance. The answers ranged from 0 (low/very easy/very dissatisfied) to 10 (extremely/very demanding/very satisfied). The following questions were answered with yes or no: “I could imagine doing a fibular construction using the MUC-Jig” and “A MUC-Jig is a practical alternative to the cutting template”. The surgeons were also able to freely comment on their perception of the methods in an empty text field. Finally, the surgeons were asked to freely suggest situations in which they consider the use of the MUC-Jig feasible or not. 

### 2.6. Statistics

Data on the participants’ demographic variables, surgeons’ subjective perception, and the operation time were evaluated by descriptive statistics.

The morphological analysis of the transplants was performed by descriptive statistics using the mean value of the triplicated measurements for each length or angle. *p*-values for relative differences were calculated using a paired two-sided t-test and for absolute differences with the Wilcoxon rank-sum test. Significance for values outside the limits (length: ±2 mm; angles: ±4°) between the four transplants and the methods was evaluated using a chi-squared test.

## 3. Results

### 3.1. Demographic Variables

A total of 10 surgeons participated in this study with an average experience in cranio-maxillofacial surgery of 11.15 y (±8.05). Detailed surgeon demographics are shown in Table 1.

### 3.2. Three-Dimensional Analysis

Both the mean relative length and the mean relative angle deviation between the MUC-Jig (−0.08 ± 1.12 mm; −0.69° ± 3.15°) and the template (0.22 ± 0.90 mm; 0.36° ± 2.56°) group differed significantly (*p* = 0.002; *p* = < 0.001). The mean absolute length and the mean absolute angle deviation of the MUC-Jig (0.81 ± 0.78 mm; 2.22° ± 2.33°) and of the template (0.69 ± 0.61 mm; 2.02° ± 1.65°) group did not differ significantly (*p* = 0.206; *p* = 0.980) 

**Figure 6:**
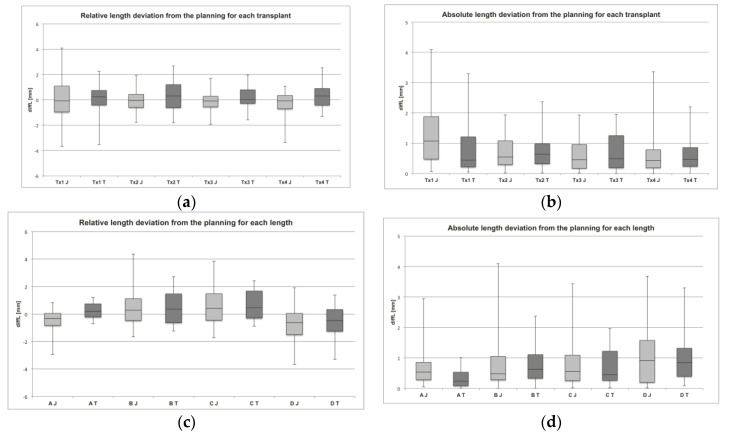
Assessed length deviations. In (**a**) and (**b**), the relative and absolute deviations from the original planning parameters are shown for each transplant (Tx1–Tx4) separately for both methods (J: MUC-Jig, T: template). In (**c**) and (**d**), the relative and absolute deviations from the original planning parameters are shown for each measured section (A–D) separately for both methods (J: MUC-Jig, T: template).

The mean length deviation (diffL) of the segments (Tx1–Tx4) (Figure 6a,b):

- Tx2 (−0.05 ± 0.89 mm) and Tx4 (−0.29 ± 0.92 mm) of the MUC-Jig group were significantly shorter compared to the template group (0.28 ± 0.83; 0.35 ± 0.74 mm; *p* = 0.024; *p* = 0.003).
- No significant relative differences (*p* > 0.05) between the segments within both groups were found.
- Tx1 (MUC-Jig: 1.28 ± 1.03 mm) showed a significant mean absolute length deviation between the groups (template: 0.78 ± 0.77 mm; *p* = 0.016) and within the MUC-Jig group (*p* ≤ 0.009) compared to the other segments (Tx2: 0.70 ± 0.54; Tx3: 0.64 ± 0.55; Tx4: 0.64 ± 0.71 mm).

The mean diffL of the sections (A–D) (Figure 6c,d):

- Section A (−0.49 ± 0.76; 0.67 ± 0.60 mm) of the MUC-Jig group showed a significant mean relative (*p* = 4.61 × 10^−7^) and absolute (*p* = 0.007) difference, as compared to the template group (0.28 ± 0.37; 0.35 ± 0.31 mm).
- Tx1–Tx4 (−0.36–−0.69 mm ± 0.65–1.01 mm) of the MUC-Jig group were significantly (*p* ≤ 0.05) shorter in section A than those of the template group (0.12–0.48 ± 0.26°–0.48°). However, only Tx1 (0.65 ± 0.48 mm) and Tx3 (0.72 ± 0.62 mm) of the MUC-Jig group also showed a significantly (*p* = 0.014; *p* = 0.049) larger absolute deviation from the planning in this section than the template (Tx1: 0.23 ± 0.16; Tx3: 0.29 ± 0.29 mm) group did.
- Sections A (−0.49 ± 0.76 mm) and D (−0.74 ± 1.24 mm) within the MUC-Jig group were shorter than the planning parameters and differed significantly (*p* ≤ 0.001) from B (0.40 ± 0.99 mm) and C (0.50 ± 0.94 mm), but not in the absolute deviation (*p* > 0.05).
- Sections A (0.28 ± 0.37 mm) and D (−0.47 ± 1.07 mm) within the template group were significantly shorter than C (0.64 ± 0.67 mm) and D (*p* ≤ 0.003) and A, B (0.44 ± 0.91 mm), and C (*p* ≤ 8.39 × 10^−6^), respectively, but did not differ significantly concerning the absolute deviation (*p* > 0.05).

**Figure 7:**
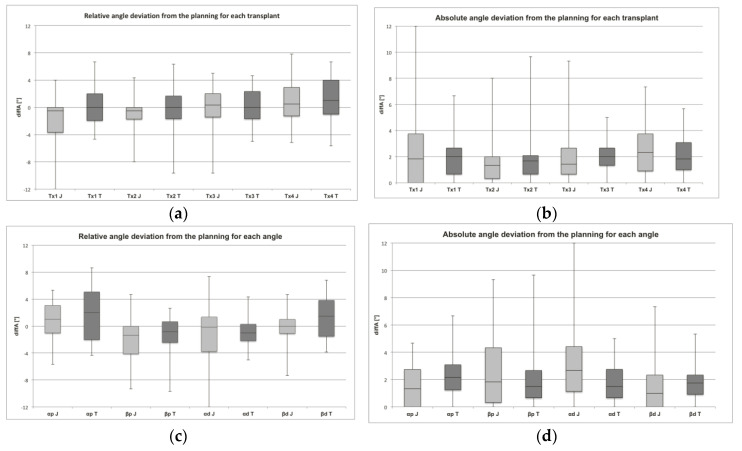
Assessed angle deviations. In (**a**) and (**b**), the relative and absolute deviations from the original planning parameters are shown for each transplant (Tx1–Tx4) separately for both methods (J: MUC-Jig, T: template). In (**c**) and (**d**), the relative and absolute deviations from the original planning parameters are shown for each measured angle (α_p_, β_p_, α_d_, β_d_) separately for both methods (J: MUC-Jig, T: template).

The mean angle deviation (diffA) of the segments (Tx1–Tx4) (Figure 7a,b):

- Tx1 (−2.04° ± 3.71°) and Tx2 (−1.07° ± 2.52°) of the MUC-Jig group showed smaller mean angles than the planning parameters and differed significantly (*p* = 0.001; *p* = 0.019) with respect to the relative deviation from the template group (Tx1: 0.05° ± 2.65°; Tx2: 0.05° ± 2.66°).
- Tx1 and Tx2 within the MUC-Jig group showed smaller mean angles compared to the planning parameters and also differed significantly (*p* ≤ 0.05) from Tx3 (−0.07° ± 2.84°) and Tx4 (0.44° ± 2.95°).
- Tx4 (1.20° ± 2.37°) also differed significantly from Tx1, Tx2, and Tx3 (0.15° ± 2.56°) within the template group in terms of a too large angle (*p* ≤ 0.05).
- No significant (*p* > 0.05) mean absolute angular deviations of the transplants between and within the groups were observed.

The mean diffA of the angles (α_p_, β_p_, α_d_, β_d_) (Figure 7c,d):

- All mean angles of the MUC-Jig group were significantly smaller (*p* ≤ 0.05) compared to the template group, with the exception of the α_d_ angle, but no significant difference (*p* > 0.05) could be found with regard to the absolute deviation from the planning at all angles.
- In both groups (MUC-Jig (J); template (T)), the variable angles (β_p_ (J: −2.06° ± 2.87°; T: −1.05° ± 2.43°), and α_d_ (J: −1.23° ± 4.22°; T: −0.89° ± 2.26°)) were, on average, smaller than the planning parameters, and at the same time they deviated significantly (*p* ≤ 0.05) from the fixed angles (α_p_ (J: 0.83° ± 1.98°; T: 2.00° ± 2.30°), and β_d_ (J: −0.28° ± 2.42°; T: 1.40° ± 1.86°)), which tended to be larger than the planning parameters.
- There were no significant absolute deviations between or within the groups (*p* > 0.05).

Looking at each measuring length and each angle separately for each Tx, it is noticeable that the significant (*p* ≤ 0.05) relative deviations within the groups were more common in the MUC-Jig than in the template group with regard to length (7 vs. 2 of 24) and angle deviation (5 vs. 4 of 24). The length deviations were always shorter compared to the planning parameters with increasing angle and distance from the stop, and the angles were always smaller than the planning parameters with increasing degrees.

Looking at these aspects with regard to the absolute deviations, only Tx1 showed significant differences at section D and angle α_d_ between the groups and within the MUC-Jig group. Tx1 showed larger angular deviations or larger length deviations compared to the other Txs of the MUC-Jig group. However, compared to the template group, the absolute length deviation in section D at Tx1 of the MUC-Jig group was scarcely significantly smaller (*p* = 0.049), but the absolute angle deviation in α_d_ was significantly larger (*p* = 0.031).

**Figure 8:**
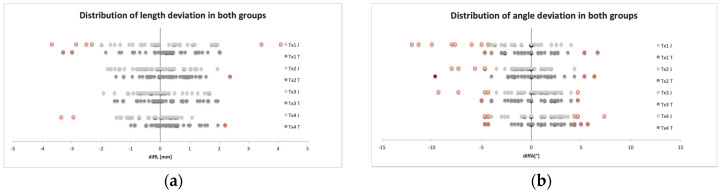
Distributions of all relative length (**a**) and angle (**b**) deviations from the original planning parameters assessed for each transplant (Tx1–Tx4) and both methods (J: MUC-Jig, T: template). Values outside the limits (±2 mm, ±4°) are shown in red.

For values outside the limits ±2 mm for length and ±4° for angle deviations from the original planning parameters:

- Limit exceedances at all angles were equally likely in both groups (Figure 8a).
- Tx1 (7 of 40) of the MUC-Jig group showed statistically significantly (*p* = 0.011) more frequent limit exceedances with respect to measured sections (A–D) than Tx2 (0 of 40) and Tx3 (0 of 40) (Figure 8b).

### 3.3. Intervention Times

The total removal (t_tot_) of the four segments took mean (SD) 20 min and 20 s (5′35″) in the MUC-Jig group and 7′19″ (1′54″) in the template group, i.e., significantly (*p* = 1.75 × 10^−5^) longer in the MUC-Jig group, with 13′01″ difference.

Consequently, the times for preparation (t_prep_) and osteotomies (t_ost_) in the MUC-Jig group (8′53″ ± 3′03″; 11′27″ ± 3′20″) were, compared to the template group (4′23″ ± 1′19″; 2′56′’ ± 0′46″), significantly longer (*p* = 0.003; *p* = 4.13 × 10^−6^), with 4′30″ and 8′31″ difference.

### 3.4. Surgeons’ Subjective Perception

**Figure 9:**
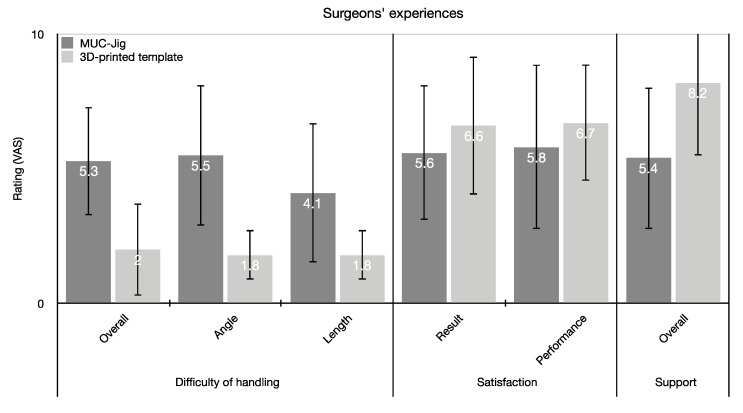
Mean values (± SD) of the surgeons’ subjective perception on a visual analogue scale (VAS). The surgeons were asked about the perceived difficulty of the methods in terms of general application, length, and angle conversion. Furthermore, the support perceived by the method and the satisfaction was also evaluated with regard to their performance and the result achieved.

The surgeons perceived the template procedure method as simpler with regard to the implementation of the cutting lengths and angles (*p* ≤ 0.05) (Figure 9). The template was also preferred for intraoperative support (*p* = 0.007). Concerning the achieved result and the own performance intraoperatively, no statistically significant (*p* ≤ 0.05) differences between the methods were found. Seven out of 10 surgeons (5/5 consultants; 2/5 residents) perceived the MUC-Jig as a valuable alternative to the template and could imagine its application in clinical routine. The most commonly described possible indication was the non-availability of the template (5/5 consultants; 3/5 residents), and as a primary application area, the surgeons mentioned simple and maximum two-segment reconstructions (4/5 consultants; 0/5 residents). Vice versa, the availability of a template or a complex geometry of the required segments was mentioned as a contraindication (4/5 consultants; 5/5 residents).

## 4. Discussion

In recent years, VSP with 3D-printed cutting guides and preformed reconstruction plates have become a well-established procedure for microvascular reconstructions, especially after segmental mandibulectomy and comprehensive reconstructions of the lower jaw. VSP allows the surgeon to plan the postoperative outcome and to fabricate the appropriate cutting guides, as well as an accurately fitting reconstruction plate, which has been demonstrated to result in better functional and anatomical outcomes compared to freehand reconstruction [13,14,15]. Besides the undeniable advantages of the VSP, the question arises whether such a procedure is always necessary, or if alternative concepts, such as reusable saw guides, which have been described repeatedly in recent years [10,16,21] and are located somewhere between the freehand method and the VSP, offer comparable intraoperative assistance for less complex reconstructions and lead to satisfactory results. Additionally, in view of the increasing healthcare costs and the desire to optimize processes in terms of associated costs while maintaining an appropriate quality, reusable saw guides also aim to reduce the planning costs of such reconstructions, which in the case of VSP, including material costs, are around USD 5000–7000 [20].

This study aimed to validate a reusable cutting jig (MUC-Jig) in terms of the precision of osteotomies performed with it on a fibula, and to assess the time needed, as well as the surgeons’ opinions of the device. All data were collected in direct comparison with a 3D-printed cutting guide.

The results show that the maximum mean deviation of both methods was within the defined limits (±2 mm, ±4°) and that neither method was inferior to the outliers of the other. The results achieved with the MUC-Jig occasionally differed from those achieved with the template at >30° angles. However, this deviation is still within limits, and it hardly differs from length [16,17,18,22,23,24,25] and angle [18,26,27] deviations of other studies. In the present study, the largest length deviations were usually located in the regions furthest away from the stop. The largest angular deviations were observed mainly at large angles and more frequently in the coronal plane. However, since these were usually too small, a correction would still have been possible. The fact that 0° cuts resulted in significant angle deviations in the template group again shows that even using a template is no guarantee for a straight saw cut.

The scattering of the data may have been smaller and the data even clearer if a piezosurgery device was used instead of the oscillating device (Colibri system), which would probably have allowed for even more precise osteotomies. However, this would have made the study’s implementation considerably more difficult, which is why the Colibri system was used.

When comparing the intervention times, the MUC-Jig is inferior to the 3D-printed cutting guide in all aspects. However, the relevance of a mean difference of 13′01′’ in a multiple-hour operation can be discussed, as reacting to an unforeseen situation is easier when using the MUC-Jig than with a template or known, reusable, universal saw templates [8,9]. However, the use of the MUC-Jig without prior VSP requires intraoperative bending of the osteosynthesis plates. Both mini plates and reconstruction plates are recommended for this [28,29]. According to the literature, the use of a reconstruction plate can cause about one hour to the operation time, which should be added to the procedure with the MUC-Jig [15,19,20].

The MUC-Jig’s economic advantages can be shown by calculating the additional costs at USD 47.50 per operation minute (USD 618.30), which results from the use of the MUC-Jig compared to the 3D-printed surgical guide [20]. Furthermore, the cost of the reconstruction plate (USD 665) and the additional time required (60–67.4 min) for intraoperative plate bending must also be taken into account (USD 2850–3201.50). Subtracting these from the cost of a VSP with 3D-printed surgical guide operation, using a pre-bent (USD 5098) or a milled plate (USD 6980), results in a difference of USD 1278.20–3160.20 in favor of the MUC-Jig. In the case of using the MUC-Jig in combination with a pre-bent plate (USD 1231.50), the associated costs would be even lower (USD 1849.80), resulting in a difference compared to VSP with a 3D-printed cutting guide using a pre-bent plate of USD 3248.20, respectively, compared with a milled plate of USD 5130.20. If the acquisition costs of the MUC-Jig of USD 1000–2000 are also taken into account, lower total costs compared to the VSP with 3D-printed surgical guides and individual reconstruction plates can be expected at the latest from the second application onwards.

In addition, preoperative planning takes little time, as it can be performed relatively simply on computed tomography images, or on the printed model for the pre-bent plate, which would require several hours of computer planning and production time for a template-guided operation. Therefore, the MUC-Jig can most probably exceed this surgical procedure in terms of the total time required for planning and conducting the operation. Moreover, as already mentioned, the MUC-Jig can be adjusted with regard to the planned parameters during surgery when the situation requires a change of the planned reconstruction while the printed guide is wasted.

As can be seen from the evaluation of the procedure, depending on the training level, that this surgical procedure seems to be particularly popular in experienced surgeons, who see it as a real alternative or at least as an emergency solution. In contrast, less experienced surgeons are more reluctant to use this method. This is probably due to the fact that younger colleagues already feel sufficiently challenged with the operation itself and welcome any help that simplifies the workflow.

As a limitation, it should be mentioned that the chosen study setup does not take into account the tight space conditions, especially those created by the soft tissue, which could not be simulated. However, it should be noted that the MUC-Jig could be applied in a pilot test on a straight shaped fibula on a surgical site. For this reason, we chose a straight fibula in our experimental setup due to the better measurability of the fibula segments. Similarly, the lengths and angles had to be analogous to the 3D-printed cutting guide to allow for one-to-one comparison of the segments harvested by the two methods. Because this study was primarily concerned with the applicability and accuracy of segment removal, intraoperative planning and the reconstruction of a mandibular defect was not performed.

Further studies are planned to demonstrate the application in patients for which this study serves as a baseline.

## 5. Conclusions

With its quite high precision, the MUC-Jig seems to be a reasonable alternative to the individually 3D-printed cutting guides widely used today in certain situations. It allows for intraoperative changes in the number, angles and lengths of the segments, which is not possible with 3D-printed guides. This is especially important in cases where the overall extent of mandibular resection cannot be accurately estimated preoperatively. Thus, in the absence or limited possibility of VSP, it can serve as a valuable tool when harvesting fibula flaps, or as method of first choice for simple, short one- or two-segment mandibular reconstructions. In addition to accuracy, economic aspects, such as universality, short planning time, and reusability, also speak in favor of the cost-effective MUC-Jig, making it particularly attractive for developing countries. Nevertheless, the MUC-Jig will not replace 3D-printed cutting guides and will remain a useful additional tool, because the technological future will belong to VSP.

## Figures and Tables

**Table 1:** Demographic variables of the participating surgeons.

	Sex		Age (y)	Experience (y)	Fibula Txs	
	*♀*	*♂*			*Performed*	*Assisted*
Consultants	1	4	47.20 (±8.87)	16.80 (±7.66)	7.40 (±8.59)	14.00 (±5.48)
Residents	1	4	35.80 (±2.49)	5.50 (±2.69)	0.00	16.40 (±14.45)
Total	2	8	41.44 (±8.59)	11.15 (±8.05)	3.70 (±6.92)	15.20 (±10.38)

## Product List:

- Multi-Use Cutting Jig (self-developed—design JM Hirsch and CS Leiggener in collaboration with Ralf Schumacher, University of Applied Sciences and Arts Northwestern Switzerland and Ångström Workshop Uppsala University, Uppsala Sweden who also manufactured the Multi-Use Cutting Jig)
- 3D-printed cutting guide (Objet30 V3 using MED610 with SUP706, Stratasys, Ltd., Eden Prairie, MN, USA)
- Planning software (Mimics Innovation Suite, V20, Materialise, Leuven, Belgium)
- Overhead camera (iPhone 7, Apple, Cupertino, United States of America)
- Colibri system (Colibri II, DePuy Synthes, Oberdorf, Switzerland)
- AO/ASIF Quick Coupling (05.001.250, DePuy Synthes, Oberdorf, Switzerland)
- Drill (310.110, DePuy Synthes, Oberdorf, Switzerland)
- Oscillating Saw Attachment (532.021, DePuy Synthes, Oberdorf, Switzerland)
- Saw blade (532.064, DePuy Synthes, Oberdorf, Switzerland).
- Screws (Spax 2 × 12 mm Senkkopf, SPAX International GmbH & Co. KG, Ennepetal, Germany).
- 3D-printer (MakerBot Replicator+, MakerBot Industries, NY, USA)
- PLA (MakerBot Filament, 1.75 mm, 0.9 kg, MakerBot Industries, NY, USA).
- Pencil (Write-4-All Permanent Marker, size F, black, STABILO International GmbH, Heroldsberg, Germany)
- Stainless steel ruler (Lineal 50 cm Stahl, Veto, Alphen aan den Rijn, The Netherlands)

## Abbreviations

3D	Three-dimensional
CMF	Cranio-maxillofacial
CNC	Computer numerical control
MAM	Medical additive manufacturing
MUC-Jig	Multi-Use Cutting Jig
PLA	Polylactic acid
*p*	*p*-value
SD	Standard deviation
Tx	Transplant
USD	United States Dollar
VAS	Visual analogue scale
VSP	Virtual Surgical Planning
